# Early hip laxity screening and later canine hip dysplasia development

**DOI:** 10.14202/vetworld.2022.679-684

**Published:** 2022-03-24

**Authors:** Ana Santana, Sofia Alves-Pimenta, Pedro Franco-Gonçalo, Lio Gonçalves, João Martins, Bruno Colaço, Mário Ginja

**Affiliations:** 1 Department of Veterinary Medicine, Lusófona University, Lisbon, Portugal; 2 CECAV Veterinary and Animal Research Centre, The University of Trás-os-Montes and Alto Douro, Vila Real, Portugal; 3 AL4AnimalS - Associate Laboratory for Animal and Veterinary Sciences, Vila Real, Portugal; 4 Department of Animal Science, The University of Trás-os-Montes and Alto Douro, Vila Real, Portugal; 5 Department of Veterinary Science, The University of Trás-os-Montes and Alto Douro, Vila Real, Portugal; 6 Department of Engineering, The University of Trás-os-Montes and Alto Douro, Vila Real, Portugal; 7 INESC-TEC – Institute for Systems and Computer Engineering, Technology and Science, Porto, Portugal

**Keywords:** canine hip dysplasia, distraction index, hip distractor DisUTAD, hip laxity

## Abstract

**Background and Aim:**

Passive hip laxity (PHL) is considered the primary risk factor for canine hip dysplasia (HD) and is estimated, in stress hip radiographs, using the distraction index (DI). The study aimed to associate the early PHL using the hip Distractor of University of Trás-os-Montes and Alto Douro (DisUTAD) and the late HD grades.

**Materials and Methods:**

A total of 41 dogs (82 hips) were submitted to a follow-up study. First, between 4 and 12 months of age, dogs were radiographed using the DisUTAD hip distractor and were determined the DI for each hip joint. Then, after 12 months of age, dogs were reevaluated for HD using the conventional hip ventrodorsal projection and hips were evaluated for HD using the Fédération Cynologique Internationale (FCI) scoring system.

**Results:**

Hips of dogs’ in the second examination with FCI grades of A (n=28), B (n=11), C (n=22), and D and E (n=21) had an early DI of 0.32±0.1, 0.38±0.08, 0.50±0.12, and 0.64±0.11, respectively. Statistical analysis using the general linear model univariate, with the DI as dependent variable and the FCI grades, side and sex as fixed factors, and the *post hoc* Bonferroni correction test showed significant differences among FCI grades (p<0.05).

**Conclusion:**

These results show the association between early DI and the late FCI HD grades and the DisUTAD is recommended for the early canine HD diagnosis.

## Introduction

Canine hip dysplasia (HD) is one of the most common non-traumatic orthopedic diseases in large and giant breeds, resulting from environmental and genetic factors [1-3]. The joint appears normal and congruent at birth, but develops abnormally during the growth of dysplastic animals [2,3]. The passive hip laxity (PHL) is the main risk factor for HD, comes early, and leads to subluxation of the femoral head, incongruity of the joint, and subsequent flattening of the acetabulum [2,4]. The pathogenic process involves an abnormal progression of endochondral ossification, followed by an inflammatory response leading to secondary degenerative joint disease, manifesting as restricted joint mobility, pain, lameness, and may justify different types of treatment or even euthanasia [2-6]. The predisposition for HD is assessed on distraction radiographs in dogs older than 16 weeks of age, measuring the distraction index (DI) [7,8] or on the standard radiographic ventrodorsal hip extended (VDHE) using signs of joint congruency and degenerative joint disease as the main references [9]. The recommended minimum age for screening HD using the VDHE is 12 months for small and medium breeds or 18 months for large and giant breeds [9,10]. For HD scoring, there are in the world several scoring systems. The Federation Cynologique Internationale (FCI) is the most popular in continental European countries and uses five scoring HD categories: A – No signs of HD; B – near-normal hips; C – mild HD; D – moderate HD; and E – severe HD. The FCI grades are defined based on values of the Norberg angle (NA), depth of acetabulum, degree of subluxation, and signs of secondary joint disease [4]. The degree of HD is essential to select the animals with the best quality of hips for breeding.

Although screening programs have been used for decades to reduce canine HD prevalence, the success is very variable in some breeds and countries [10-13]. When prevalence and severity of HD reduce, the identification of slightly affected animals needs more refined selection tools to obtain more progress [11] and the PHL can give additional medical information. The PHL allows an early exclusion from breeding or training programs and enables early HD management decision in growing dogs [14,15]. However, the thresholds and variations of the early DI for the prediction of the late FCI grade remain undefined [16]. A hip distraction view is performed to demonstrate PHL using a custom-designed device distractor, enabling then the calculation of the DI [8]. There are at least three hip joint distractors, the PennHIP [8], the Vezzoni-modified Badertscher distension device, produced by Fondazione Salute Animale, Cremona, Italy [7,17], and the hip Distractor of University of Trás-os-Montes and Alto Douro (DisUTAD) [18], which have been used to obtain the distraction views. The DisUTAD was recently developed, trying to improve some functional technical aspects of the use and hip distraction [18,19]; it is intended that it will be available to interested veterinarians in the near future.

This study aimed to associate the early PHL using the DisUTAD hip distractor and the late HD grades.

## Materials and Methods

### Ethical approval

All examinations were performed with the owner’s consent and in compliance with the regulations of our institutions (Approval no. 1044-e-DCV-2018) and in accordance with the European regulations for animal use and care (European Directive 2010/63/EU and National Decree-Law 113/2013).

### Study period and location

The study was conducted from January 2015 to December 2020 at the Veterinary Teaching Hospitals of Trás-os-Montes and Alto Douro (UTAD) and Lusófona Universities, Portugal

### Animals

This was a prospective study and 41 dogs (82 hip joints) were included in the study. Each dog was presented twice at the Veterinary Teaching Hospitals of UTAD and Lusófona University. At the first radiographic examination, dogs were older than 4 months and younger than 12 months of age, and at the second, dogs were older than 12 months of age; the minimum period of time between both examinations was 6 months. At the first examination, the dogs were screened for PHL measuring the DI and at the second examination were evaluated for HD using the FCI scoring system. Recorded data included the breed, sex, age, and body weight at the time of each radiograph. The inclusion criteria required normal musculoskeletal development in the clinical examination. The sample size was determined for statistical significance of 0.05, an effect size of 0.5, and a power of 0.8, which require a minimum sample of 80 observations [20].

### Radiographic procedure and measurements

Radiographs were acquired with dogs under deep sedation using dexmedetomidine (0.02 mg/kg i.v.) (Dexdomitor: Orion Corporation, Espoo, Finland) and butorphanol (0.1 mg/kg i.v.) (Torbugesic Injectable: Fort Dodge Veterinaria, Girona, Spain). Sedation was reversed with atipamezole hydrochloride (0.02 mg/kg i.m.) (Antisedan: Orion Corporation).

The hip distraction view was obtained with dogs in dorsal recumbency at the first examination using DisUTAD (UTAD, Vila Real, Portugal). The DisUTAD is a trapezoidal-shaped rubber hip distractor developed at UTAD that promotes adequate hip distraction in dogs [18,19]. Hip distraction was achieved following the technique originally described in previous work [18]. At the second examination (minimum 12 or 18 months of age), dogs underwent the VDHE view and the HD assessment in accordance with FCI standards. The VDHE view was obtained with the dog in dorsal recumbency on the X-ray table, with femurs parallel, extended, and internally rotated [19].

The DI measurements on the first examination were performed for each hip joint in the distraction radiograph, using the dedicated Dys4Vet software (Neadvance Machine Vision SA, Braga, Portugal) and a methodology previously described [20]. The DI was obtained as described previously, dividing the lateral femoral head displacement by its radius; a DI of 0 represents absolute joint congruity while a DI around 1 represents a higher hip laxity [8,16]. At the second examination, each joint was assigned one of the five grades (A-E), defined using the FCI guidelines, which include the NA degrees laxity [2,21]. The NA was determined using the Dys4Vet software and was measured between a line that connects the center of the right and left femoral head and another connecting the center of the femoral head and the other tangent to craniolateral margin of acetabular rim [21]. The DI measurements, NA calculations, and FCI scores were performed by AS, PF, and MG, respectively.

### Statistical analysis

Data analysis was performed using the Statistical Package for the Social Sciences version 27 statistical software (IBM Corp., Armonk, NY, USA). The assumption of normal distribution of DI in the sample was assessed using Shapiro–Wilk test. For statistical purposes, the general linear model univariate with the Bonferroni correction *post hoc* test was used in data analysis considering DI as the dependent variable, and the FCI hip organized in four groups (A, B, C, and DE), the sex (male and female), and the side (right and left) as fixed factors. The null hypothesis was that there is no difference in mean DI among groups of studied fixed factors and the alternative hypothesis was that these means were not equal [22]. The linear Pearson correlation was used to evaluate the association between the early DI and the later NA and coefficient of determination to evaluate the proportion of the variance in the NA that is predictable from the DI. Significance was reported when p<0.05 [23].

## Results

A total of 41 dogs of four breeds were used in the study; the most frequently represented breed was the Estrela Mountain dog (24 dogs, 58.5%) and the other dogs were of two large (Transmontano Mastiff and Alentejo Mastiff) and one medium breed (Portuguese Pointer Dog), making together 17 animals (41.5%). Twenty-three dogs were female and 18 males. The DI had a normal distribution in the Shapiro–Wilk test (p=0.89).

**Figure-1:**
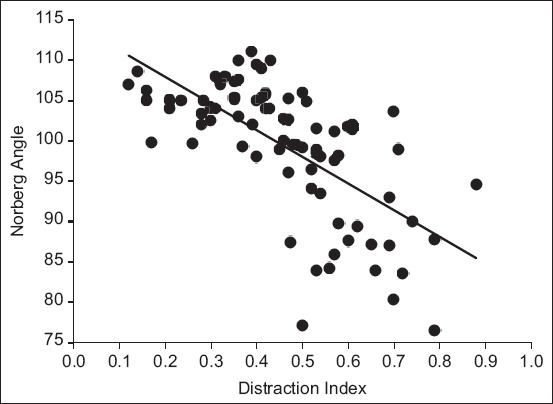
Scatterplot of early distraction indices versus later Norberg angle in degrees.

At the first evaluation, the dogs’ age ranged from 4 to 11 months, mean±standard deviation (SD) 6.6±2.2 months, and bodyweight ranged from 15 to 45 kg (26.4±8.3 kg). In the second evaluation, the ages ranged from 13 to 48 months (21.4±7.1 months) and body weight ranged from 17 to 65 kg (39.7±12.2 kg). The period of time between both examinations ranged from 6 to 44 months (14.8±7.6 months). Early DI ranged from 0.12 to 0.88 (0.46±0.17) and the late NA ranged from 76.5 to 111° (99.3±8.2°). The linear Pearson correlation between the early DI and the late NA was −0.66 and the coefficient of determination was 0.44 (p<0.001) (Figure-1).

**Figure-2:**
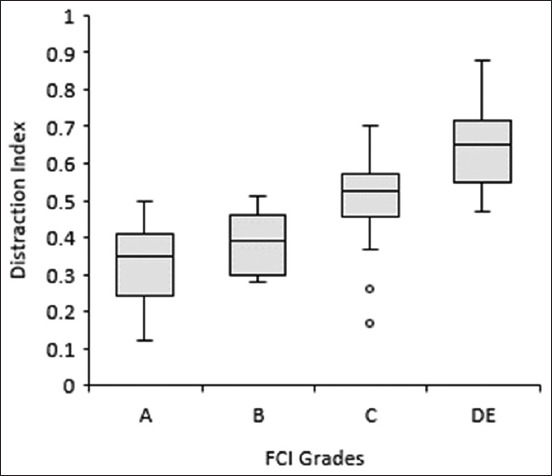
Box-and-whisker plot presenting the early distraction index of hip joints classified in late different Fédération Cynologique Internationale grades (a-e).

The general linear model univariate analysis showed significant differences in DI of the FCI groups (p<0.001) and non-significant on side (p=0.85), sex (p=0.77), and interaction groups (p=0.27 to 0.85). The FCI hips Grade A (n=28) presented an early mean±SD DI of 0.32±0.1, B (n=11) DI of 0.38±0.08, C (n=22) DI of 0.50±0.12, and DE (n=21) LI of 0.64±0.11 (Table-1 and Figure-2). The null hypothesis was accepted for the mean DI on sex and side and rejected for FCI grades.

**Table-1:** Distraction indices of hip joints at the first radiographic evaluation, grouped by FCI grades at the second evaluation, sex and side.

Group	Hips number	Distraction index first evaluation	

		Mean±SD^1^	Range
FCI grades second evaluation			
A	28	0.32±0.1^a^	0.12–0.50
B	11	0.38±0.08^a^	0.28–0.51
C	22	0.50±0.12^b^	0.17–0.70
D and E	21	0.64±0.11^c^	0.47–0.88
Sex			
Male	36	0.46±0.17^a^	0.12–0.79
Female	46	0.46±0.17^a^	0.16–0.88
Side			
Right	41	0.46±0.16^a^	0.14–0.74
Left	41	0.46±0.18^a^	0.12–0.88

FCI=Fédération Cynologique Internationale, SD=Standard deviation.

1 Mean values with different superscripts in the same row and group are significantly different (p<0.05) using the general linear model univariate test; the FCI groups’ differences were evaluated using the Bonferroni correction *post hoc* test.

## Discussion

The objective of this study was to associate the early DI, demonstrated using the DisUTAD distractor, to the hip quality of mature hips classified for HD using FCI scoring system. Our results showed that different FCI Groups A, B, C, and DE have early DI means gradually higher with differences statistically significant (p<0.05) that allows us to reject the null hypothesis with a power of 80% [22]. The moderate negative (−0.66) linear Pearson correlation between early DI and late NA, resulting in a coefficient of 0.44, indicates that 44% of the variance of NA in adult animals is associated with the early DI [23]. The negative correlation coefficient was also expected since a higher early DI results in greater instability and in the development of a shallower acetabulum, which reduces the NA [24]. These results agree with similar studies using other hip distractors [16,24,25]. The option of putting together FCI Grades D and E intended to make the number of animals by groups more homogeneous and became the statistical analysis more consistent. The strategic reorganization of similar FCI categories for statistical purposes has already been carried out in the previous works to improve the research results [2,4,13,25]. The influence of breed, age, and body weight was not investigated because of the reduced number of dogs of some breeds and due to the large divergence in dog’s body weight and age and their non-normal distribution.

The significant statistical differences between FCI grades (normal or near-normal hips vs. dysplastic grades) were more marked than in previous work [25] and can be attributed much clinical importance. The difference between DI of FCI Grades A and B was not significant; similar results were also described in other works, performed with other distractors and breeds [16,25]. However, the bigger DI of Grade B (0.38) than A (0.32) is interesting data, which can result in significant differences with a larger sample. The differences between FCI Grades A and B in our study were bigger than DI differences in a previous work, B (0.36) and A (0.32), which were significant [16]. Overall and despite some dog breed variations for PHL, the DI of less than 0.3 is considered the reference value for a normal hip development [8], although there may be normal hip development with DI >0.30 [2,16,24]. In Figure-1, other exceptions are also evident, and small initial DI results in joints NA around 100° characteristic of mild dysplasia [2]. The similar DI between the right and left sides agrees with the previous study [26]. However, this last study also reported a bigger mean DI in female joints than in male joints that were not registered in our work [26]. As most studies indicate a similar prevalence of HD in males and females, it is also expected that its major early risk factor, the DI be similar in both sexes [2]. Clinical methodologies, such as the Ortolani maneuver, to predict FCI scores, are more effective at a slightly older age (around 9 months) [27].

The hip congruence demonstrated by the VDHE view may also justify the moderate negative linear correlation (−0.66) between the early DI and the later NA. A greater correlation and determination coefficient could be expected if the NA will be an ideal indicator of hip laxity or degenerative joint disease development and if the NA did not depend on other dogs hip morphological or positioning factors [8,21,28]. The VDHE view is common in official screening programs worldwide. Several studies associated this technique with a lack of sensitivity to diagnose hip laxity and provide few clues for the later development of osteoarthritis [8,24,29]. This lack of sensitivity is attributed to the positioning of the dog for the VDHE view. The extension of femurs results in torsion of the joint capsule and surrounding soft tissues that favor artificial hip congruence [8]. To improve the identification of hip joint laxity, distraction-based radiographic techniques are essential [24]. This could detect false-negative animals and overcome inherent limitations in recent screening programs with a poor database of dog population that did not allow the accurate use of breeding values for selection purposes [24]. Despite all the criticism about the use of NA as a determining measure in the classification of HD, it remains an important objective reference for FCI and other HD scoring systems, with a very comprehensive recognition.

The overlap of early DI values of some late dysplastic and non-dysplastic FCI grades may also in part be explained by different pelvic musculature development in some breeds. Muscular hip mass can compensate the early PHL, delaying the development of a dorsal acetabular rim slope and the functional or active laxity, which accelerate the development of degenerative joint disease. A study also refers certain oscillation of the DI during the joint development (4-12 months of age) [30]. A significant number of dogs have also been shown signs of osteoarthritis after 5 years of age [28].

The best methods for reducing canine HD prevalence are those with the ability to detect affected animals that allow to remove them from breeding. The recommended strategies involve hip laxity measurement and calculation of estimated breeding values (EBVs), offspring control and genomic selection [4]. In the absence of laboratory diagnostic testing, radiographic examination remains the current method [15,28]. Leighton *et al*. [13] explained the importance of incorporating DI into EBV calculations for more rapid hip status improvement.

New hip distractors may allow a complete and adequate in-house evaluation of the hip joint laxity by trained clinicians and might increase the popularity of laxity-based radiographic techniques [17]. This study shows that PHL demonstrated using DisUTAD hip distractor can be a reliable method for early prediction of late canine HD and enable preventive HD measures to be employed. However, this study is associated with limitations mainly due to the low number of animals and breeds included, making it difficult to use a more comprehensive and robust statistical analysis studying the influence of other factors on DI and HD, such as the breed, body weight, and age.

## Conclusion

It can be concluded that the DisUTAD hip distractor can be used to demonstrate PHL, for early hip laxity examination and prediction of late FCI HD grades in Estrela Mountain Dog, Transmontano Mastiff, Alentejo Mastiff, and Portuguese Pointer Dog breeds. However, the low number of animals and breeds included is an important limitation. Similar studies in other breeds with a larger sample, are recommended using the DisUTAD to obtain more robust results.

## Authors’ Contributions

AS, SA, JM, PF, and MG: Carried out dog`s radiographic examinations and image measurements. BC, LG, and MG: Performed the analysis of data. AS and SA: Drafted the manuscript. MG: Revised the manuscript. All authors have read and approved the final manuscript.
